# Molecular and biochemical characterization of *Entamoeba histolytica* fructokinase

**DOI:** 10.1007/s00436-015-4383-5

**Published:** 2015-02-21

**Authors:** Julia Matt, Michael Duchêne

**Affiliations:** Institute of Specific Prophylaxis and Tropical Medicine, Center for Pathophysiology, Infectiology and Immunology, Medical University of Vienna, Kinderspitalgasse 15, 1090 Vienna, Austria

**Keywords:** *Entamoeba histolytica*, Glucose, Fructose, Fructokinase, Hexokinase

## Abstract

*Entamoeba histolytica* is the causative agent of amoebic dysentery and liver abscess. The medium for its axenic culture contains glucose as energy source, and we addressed the question whether *E. histolytica* can also use fructose instead. As the amoebic hexokinases do not phosphorylate fructose, a separate fructokinase is essential. The genome project revealed a single candidate gene encoding an *E. histolytica* homolog of bacterial fructokinases. This gene was cloned, and the recombinant enzyme had a magnesium-dependent fructose 6-kinase activity (EC 2.7.1.4) with a *K*_m_ for fructose of 0.156 mM and a *V*_max_ of 131 U/mg protein. Recombinant fructokinase also showed a much weaker mannokinase activity, but no activity with glucose or galactose. The amoebae could be switched from glucose to fructose medium without any detectable consequence on doubling time. Fructokinase messenger RNA (mRNA) was modestly but significantly upregulated in amoebae switched to fructose medium as well as in fructose-adapted *E. histolytica*.

**Electronic supplementary material:**

The online version of this article (doi:10.1007/s00436-015-4383-5) contains supplementary material, which is available to authorized users.

## Introduction

The protozoan parasite *Entamoeba histolytica* is the cause of amoebic dysentery and liver abscess. In an older study (Walsh 1986), between 36 and 50 million cases of disease and up to 110,000 deaths per year were estimated, whereas in a recent assessment of the situation in 2010, the disease burden of amoebiasis was estimated at 2.24 million disability-adjusted life years (DALYs) lost annually (Hotez et al. 2014).

The intestinal parasite exists in a microaerophilic environment and lacks a functional Krebs cycle, mitochondria and oxidative phosphorylation, so glycolysis is the major source of energy (Reeves 1984). Accordingly, the two most commonly used media for the axenic culture of *E. histolytica*, TYI-S-33 (Diamond et al. 1978) and YI-S (Diamond et al. 1995) both contain glucose as the major energy source. Glucose is readily taken up (Serrano and Reeves 1974) and phosphorylated by one of the two hexokinases (Ortner et al. 1995) as the first step of glycolysis. These two steps together with glycogen breakdown were found to have the largest influence on the glycolytic flux (Pineda et al. 2014).

In the human host, under normal conditions, almost 100 % of the glucose is absorbed before it reaches the colon and the amoebae never encounter the glucose concentration provided in the culture media. In contrast, fructose may be found at varying but sometimes significant concentrations, at least in case of fructose malabsorption, which is a common trait (Latulippe and Skoog 2011).

Although *E. histolytica* can tolerate only modest oxygen concentrations, the organism is able to consume oxygen and its uptake is strongly stimulated by glucose (Weinbach and Diamond 1974). The glycolytic pathway from glucose to acetyl-CoA generates NADH. To regenerate NAD^+^, NADH can be used to reduce acetyl-CoA to ethanol, or NADH can be transformed to NADPH which can reduce oxygen eventually to H_2_O. Thus, acetyl-CoA can be spared for the generation of an extra molecule of ATP, and this type of aerobic metabolism provides a small benefit for the amoebae.

In *E. histolytica*, fructose stimulated the aerobic metabolism at only 30 % of the level of glucose (Weinbach and Diamond 1974); moreover, the two hexokinase isoenzymes of *E. histolytica* were unable to phosphorylate fructose (Kroschewski et al. 2000), unlike the hexokinases of the human host (Middleton 1990). On the other hand, the *E. histolytica* genome (Loftus et al. 2005) contains a gene coding for a sugar kinase with similarity to bacterial fructokinases. It was hypothesized that this gene was acquired from bacteria by lateral gene transfer (Loftus et al. 2005).

In general, sugar kinases can be grouped into non-homologous families: Two large families of hexokinases and ribokinases plus a smaller family of sugar kinases with substrate binding regions in common with homoserine kinases were defined (Bork et al. 1993). Later, a fourth family of receptor kinases (ROK), which also comprises sugar kinases, was added (Titgemeyer et al. 1994). The *E. histolytica* fructokinase belongs to the ribokinase family. In the NCBI protein database (http://www.ncbi.nlm.nih.gov/protein/), homologs of the *E. histolytica* fructokinase are present in *Entamoeba nuttalli*, *Entamoeba dispar*, and *Entamoeba invadens*. A BlastP search (http://blast.ncbi.nlm.nih.gov/Blast.cgi) showed that the closest relatives outside the genus Entamoeba were from *Prevotella* spp.; one of these gene products was characterized as a fructose 6-kinase (EC 2.7.1.4) (Fuse et al. 2013).

In the present study, we investigated whether *E. histolytica* is able to grow in a medium with fructose replacing glucose and if this medium switch would cause an upregulation of the putative fructokinase gene on the messenger RNA (mRNA) level and on the level of enzyme activity. The *E. histolytica* fructokinase was expressed in *Escherichia coli*, and its substrate specificity and kinetic parameters were measured. Finally, the enzyme was localized by confocal immunofluorescence.

## Materials and methods

### Microorganisms

*E. histolytica* trophozoites (HM-1: IMSS) were grown axenically in TYI-S-33 medium (Diamond et al. 1978) containing 10 % (*v*/*v*) bovine serum at 37 °C. The cells were harvested after 48 h of incubation by centrifugation at 500 × *g* for 5 min, followed by two washings with phosphate-buffered saline (PBS). For experiments performed with fructose-adapted amoebae, the harvested trophozoites were transferred to medium containing 10 g/l (55.5 mM) fructose, instead of 10 g/l (55.5 mM) glucose. Fructose-adapted cultures remained viable for at least 12 months.

*E. coli* strain INVαF′ [F′ *end*A1 *rec*A1 *hsd*R17 (r_k_^−^,m_k_^+^) *sup*E44 *thi*-1 *gyr*A96 *rel*A1 Φ80*lac*ZΔM15 Δ(*lac*ZYA-*arg*F) U169 λ^−^] (Invitrogen, Life Technologies) was used for the direct cloning of the PCR-amplified fructokinase gene. *E. coli* strain BL21-AI [F^−^ *ompT hsdS*_B_ (r_B_^−^m_B_^−^) *gal dcm araB*::*T7RNAP-tetA*] from the same provider was used for protein expression.

### Cloning and recombinant expression of *E. histolytica* fructokinase

The coding sequence from the *E. histolytica* intronless fructokinase gene (XM_646995) was amplified by PCR from genomic *E. histolytica* DNA which was prepared using the DNeasy Blood and Tissue Kit (Qiagen). The primers 5′-CCG GCT AGC ATG AAC CAT AAA AAA ATT AAA GTA G-3′ and 5′-CAT CCA GCT CGA GTT AGT GAT GGT GAT GGT GAT GTT TTA ACT CAG ATA AAA GCT C-3′ were used for amplification. PCR was performed with Phusion High-Fidelity DNA Polymerase (Thermo Scientific), and the resulting fragment was gel-purified with the QIAquick Gel Extraction Kit (Qiagen) and cloned into the vector pCR II using the TA Cloning Kit Dual Promoter (Invitrogen, Life Technologies). The nucleotide sequence was checked by sequence analysis (Microsynth, Balgach, Switzerland). After digestion with EcoRI, purification was performed with the QIAquick PCR Purification Kit, and the product was ligated into the pET-17b vector (Novagen).

For expression, the plasmid was transformed into BL21-AI competent *E. coli*. Induction was performed at OD_600_ = 0.4 with 0.5 mM isopropyl β-D-1-thiogalactopyranoside (IPTG) and 0.2 % (*w*/*v*) arabinose, followed by 4 h culture at 37 °C. Cells were harvested by centrifugation at 5000 × *g* for 10 min at 4 °C, resuspended in native lysis buffer containing 50 mM NaH_2_PO_4_, 300 mM NaCl, 10 mM imidazole, 100 μg/ml lysozyme, pH 8.0, and disrupted in a mortar. The crude lysate was centrifuged at 18,000 × *g* at 4 °C to remove debris, and the recombinant protein in the supernatant with a predicted molecular mass of 33.6 kDa was purified under native conditions using Ni-NTA Spin Columns (Qiagen), and the obtained fractions were analyzed by 12 % SDS-polyacrylamide gel electrophoresis (SDS-PAGE).

### Quantitative reverse transcription PCR (qRT-PCR)

The expression levels of fructokinase mRNA were examined by qRT-PCR of amoebae either grown in normal medium containing glucose or of amoebae adapted to fructose for 4 weeks. Moreover, expression levels of trophozoites freshly switched to fructose medium for a total of 2 or 4 h were investigated.

Total RNA extraction was performed with the GeneJET RNA Purification Kit (Thermo Scientific). The RevertAid First Strand cDNA Synthesis Kit (Thermo Scientific) was used for reverse transcription. The reaction using a final total RNA concentration of 25 ng/μl was run at 42 °C for 70 min followed by 6 min inactivation at 70 °C. For dilution series, 5 μl aliquots were diluted 1:10 and used in qRT-PCR, always carried out in duplicate. The master mix consisted of 3.5 mM MgCl_2_, 1 × PCR-buffer B2 (Solis Biodyne), 0.2 mM dNTP mix (Thermo Scientific), 0.8 × Eva Green Dye (Biotium), and 1U HOT FIREPol DNA Polymerase (Solis Biodyne). To a final reaction volume of 20 μl, 2 μl template cDNA and 250 nM each (final concentration) of forward and reverse primer were added. For primer design, the program Primer3Plus (http://www.bioinformatics.nl/cgi-bin/primer3plus/primer3plus.cgi) was used, and the primers were checked for secondary structures (http://mfold.rna.albany.edu/?q=mfold/dna-folding-form) and dimers (http://www.premierbiosoft.com/netprimer/). Primer-BLAST (http://www.ncbi.nlm.nih.gov/tools/primer-blast/) was used to check for pseudogenes or other homologs. The sense and antisense primers were the following: 5′-GGT GAG GTT GTT TGG GAT TG-3′ and 5′-TTC CAA CAG CAA TGA AAG CA-3′. qRT-PCR was carried out with the Roche Light Cycler 480 II using the following protocol: 95 °C for 15 min, 45 cycles of (95 °C, 15 s, 60 °C, 30 s, and 72 °C, 20 s), and a final extension at 72 °C for 10 min. Experiments were performed in triplicate, and positive and negative controls were included in each run. Hexokinase 2 (XM_650873) was used as reference gene, and statistical analysis was performed with the program REST (“relative expression software tool”), available at http://rest.gene-quantification.info/ (Pfaffl et al. 2002).

### Kinetic parameters and substrate specificity

The assay for analysis of fructose phosphorylation activity measured the ADP generated in the fructokinase reaction (ADP assay). The decrease of NADH in the coupled lactate dehydrogenase reaction was examined spectrophotometrically at 340 nm. The standard assay mixture (slightly modified from Kroschewski et al. (2000)) contained 1 mM fructose, 2 mM ATP, 100 mM KCl, 10 mM MgCl_2_, 0.2 mM NADH, 0.4 mM phosphoenolpyruvate, 6 U/ml pyruvate kinase (from rabbit muscle, Sigma-Aldrich), 6 U/ml lactate dehydrogenase (from porcine heart, SERVA), and 50 mM Tris–HCl pH 8.0. Ten micrograms of recombinant *E. histolytica* fructokinase was added to a total volume of 1 ml, and the reaction was monitored over a time period of 1 min. To optimize the reaction, various pH (pH 6–9) and temperature conditions (RT, 37 °C) were tested at various fructose concentrations (0.005–10 mM). Measurements with the addition of MnCl_2_ or CaCl_2_ (10 mM) and in the absence of MgCl_2_ were also carried out. Moreover, the putative phosphorylation of glucose, mannose, and galactose (starting concentration: 5 mM) by the recombinant fructokinase was examined. All experiments were carried out in triplicate, and mean values were used for analysis. *K*_m_ and *V*_max_ were calculated with the software GraFit, Version 7 (Erithacus Software Ltd., UK), using the non-linear curve fitting program.

To analyze if the product of the fructokinase reaction was fructose 6-phosphate, the assay was coupled to the phosphoglucose isomerase and glucose-6-phosphate dehydrogenase reactions, and NADPH formation was measured. The assay mixture contained 1 mM fructose, 2 mM ATP, 10 mM MgCl_2_, 50 mM Tris–HCl pH 8.0, 0.2 mM NADP^+^, 6 U/ml glucose-6-phosphate dehydrogenase (from baker’s yeast, Sigma-Aldrich), 0.1 U/ml phosphoglucose isomerase (from baker’s yeast, Sigma-Aldrich), and 10 μg of recombinant fructokinase.

Moreover, fructokinase activity was examined in lysates of *E. histolytica* cells via measurements of NADPH formation, using the same assay mixture as above. The activity was measured in amoebae either grown in fructose or in glucose. For lysate preparation, trophozoites were washed in PBS twice, resuspended in extraction buffer (50 mM Tris–HCl, 1 mM EDTA, pH 7.4), and broken with a Dounce homogenizer. After centrifugation at 14,000 × *g* for 5 min, protein concentration of the supernatant was determined with the Bradford assay (Bio-Rad) and 500 μg of total lysate proteins were used per reaction.

### Immunofluorescence assay

*E. histolytica* trophozoites were cultured and fixed in 4-well μ-Slides (ibidi, Martinsried, Germany). Seven hundred microliters of cell suspension was pipetted into the wells, and the slides were incubated at 37 °C for 2 h in a box containing Anaerocult A (Merck). The following steps were all carried out at room temperature. *E. histolytica* cells were fixed with 4 % (*w*/*v*) paraformaldehyde (Sigma-Aldrich) in PBS for 20 min, followed by a washing step with PBS. Afterwards, the cells were incubated for 10 min with 50 mM ammonium chloride (Sigma-Aldrich) and washed with PBS twice. Incubation with 0.1 % (*w*/*v*) saponin (Sigma-Aldrich) in PBS and mouse antiserum (Davids Biotechnologie, Regensburg, Germany) 1:500 was performed for 1 h. As negative control, pre-immune serum was used. After three washings with PBS, amoebae were stained with Alexa Fluor 488 goat anti-mouse IgG (Invitrogen, Molecular Probes) 1:1000 in PBS for 30 min in the dark. Then, three washing steps followed, and to stain the nuclei, 5 min of incubation with 4′,6-diamidino-2-phenylindole (DAPI) (Sigma-Aldrich) 1:2000 in water was performed. After three more washings, cells could be stored in PBS in the dark. Microscopy was carried out with the LSM 700 confocal microscope (Carl Zeiss, Germany), and pictures were analyzed with ZEISS Efficient Navigation (ZEN) imaging software.

## Results

### *E. histolytica* trophozoites can be cultured in medium containing fructose

For all experiments with fructose-adapted amoebae, cells were grown in medium containing 10 g/l fructose instead of 10 g/l glucose for 4 weeks. Until now, after more than 12 months, the amoebae continue to proliferate well in the fructose medium.

### Cloning and recombinant expression of the *E. histolytica* fructokinase

**Fig. 1:**
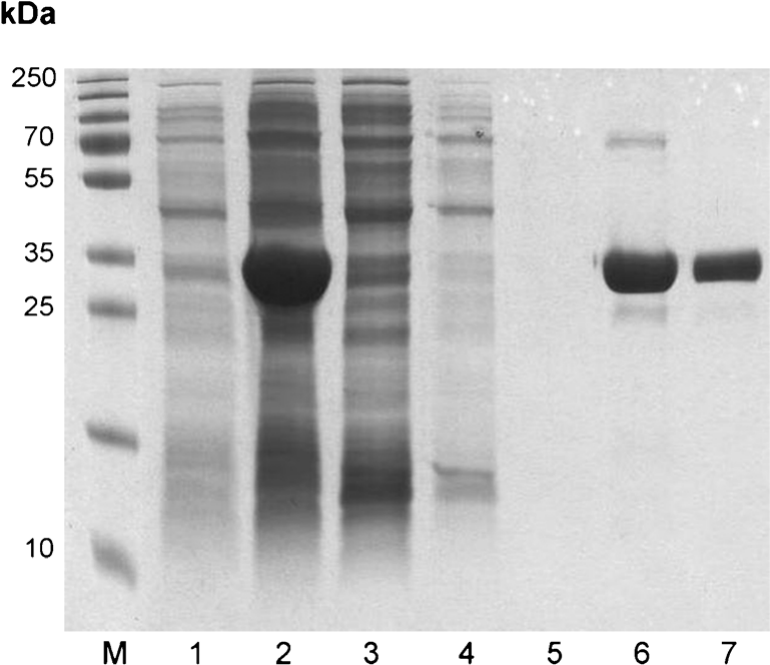
SDS-PAGE showing the purification of recombinant *E. histolytica* fructokinase*. Lane 1 E. coli* lysate from non-induced cells, *lane 2* lysate from arabinose and IPTG-induced cells, *lane 3* flow-through fraction after binding of proteins, *lanes 4–5* washing fractions, *lanes 6–7* elution fractions containing the purified recombinant fructokinase. Marker proteins are shown on the left side (M)

As the *E. histolytica* hexokinases are unable to phosphorylate fructose (Kroschewski et al. 2000), the putative fructokinase was studied. Its open reading frame (XM_646995) was amplified by PCR, and the resulting fragment was engineered into the pET-17b vector. The protein was expressed abundantly (around 15 μg/ml) in *E. coli* BL21-AI cells from which it was purified under native conditions using Ni-NTA Spin Columns. Stored at −20 °C in 50 % (*v*/*v*) glycerol, the enzyme was stable for at least 6 months. SDS-PAGE analysis of the purified protein revealed a band with an apparent molecular mass of slightly below 35 kDa, corresponding to the calculated molecular mass of 33.6 kDa including the hexahistidine tail (Fig. 1).

### Upregulation of the fructokinase mRNA in amoebae switched to fructose medium

**Fig. 2:**
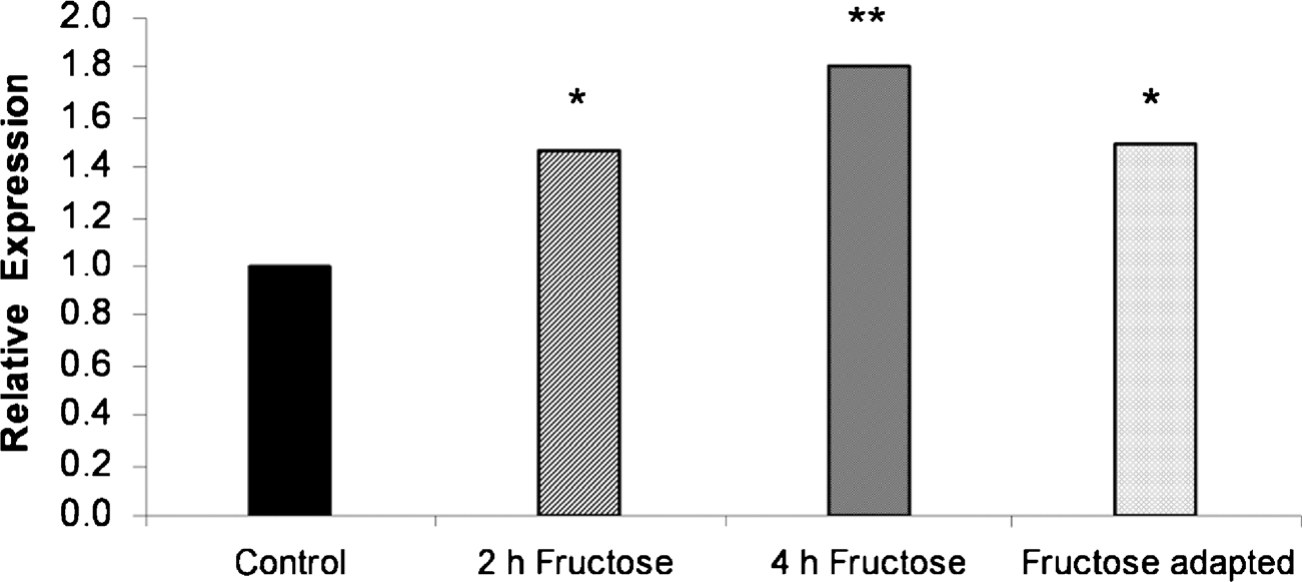
*E. histolytica* fructokinase mRNA expression of amoebae grown in medium containing fructose. *E. histolytica* trophozoites grown in fructose for 2 h upregulated fructokinase mRNA by the factor 1.47 (*p* < 0.05). Highest expression ratios were observed after 4 h of cultivation in fructose medium with a mean upregulation of 1.81 (*p* < 0.005). Compared to cells grown in normal medium, fructose-adapted amoebae showed an upregulation of 1.5 (*p* < 0.05)

*E. histolytica* trophozoites were switched from 10 g/l glucose to 10 g/l fructose medium. Total RNA was extracted from the original culture and after 2 and 4 h in fructose medium. After reverse transcription, the relative expression of fructokinase mRNA was determined by qRT-PCR (Fig. 2). In general, only a modest upregulation of fructokinase expression was observed, 1.47-fold after 2 h (*p* < 0.05) and 1.81-fold after 4 h (*p* < 0.005). Compared to amoebae grown in glucose medium, the expression of fructokinase mRNA in amoebae grown in fructose medium for 4 weeks remained elevated 1.5-fold (*p* < 0.05). The efficiency of amplification was between 0.85 and 1.

### Kinetic parameters and substrate specificity of *E. histolytica* fructokinase

**Fig. 3:**
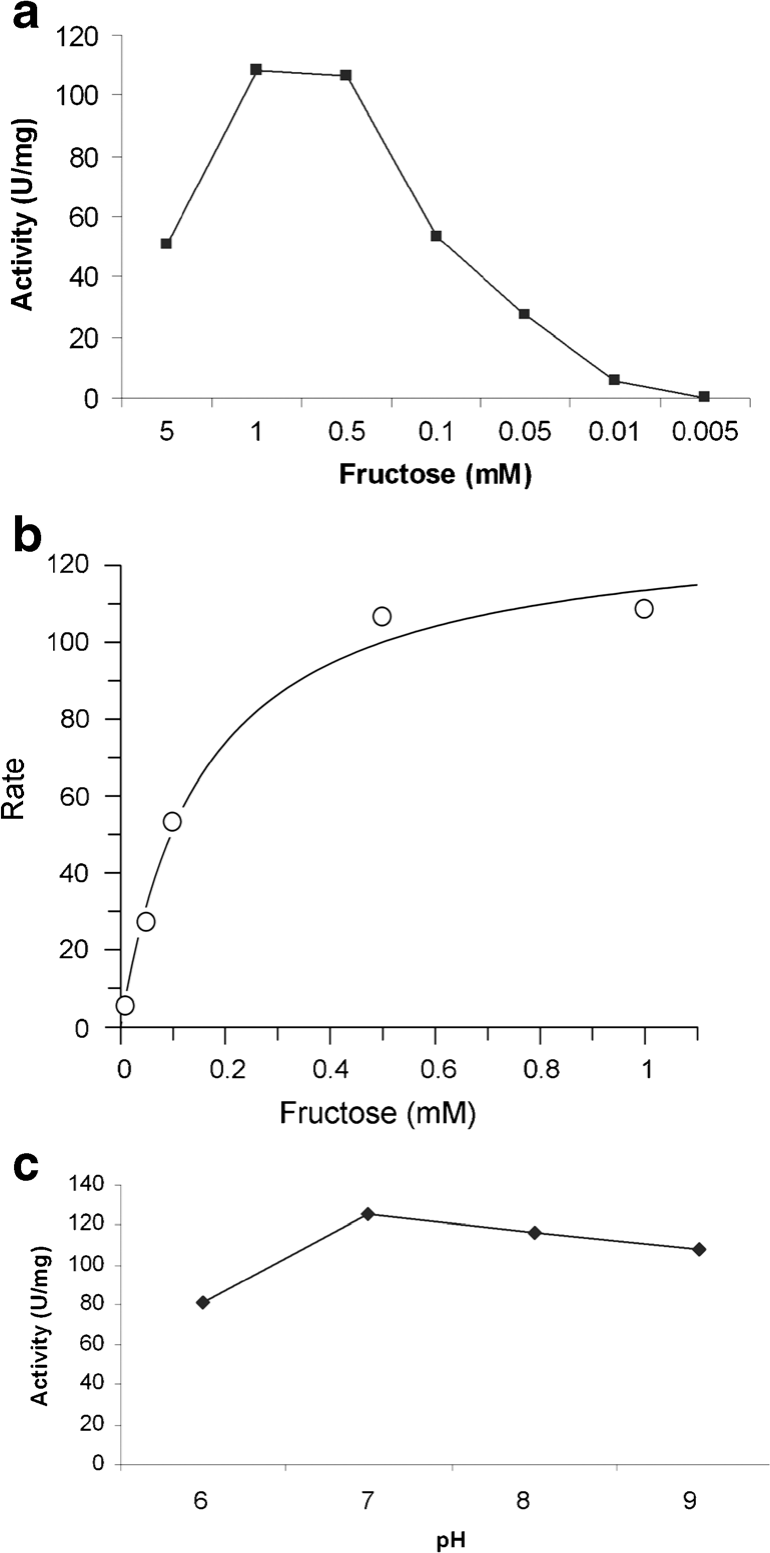
**a** Fructose phosphorylation activity in the fructokinase reaction with different substrate concentrations. Generated ADP was measured spectrophotometrically via the decrease of NADH in the coupled lactose dehydrogenase reaction, at the temperature optimum of 37 °C. Highest fructokinase phosphorylation activity was observed with 1 mM fructose (108.5 U/mg). Similar results were observed with 0.5 mM fructose, whereas the activity decreased using lower concentrations. At 5 mM fructose concentration, the activity decreased to about half of that observed at 1 mM. **b** Fructokinase reaction rate as a function of fructose concentration (0.01–1 mM). Calculated *V*_max_ of *E. histolytica* fructokinase at 37 °C was 131.25 U/mg protein and *K*_m_ was 0.156 mM. **c** pH dependency of *E. histolytica* fructokinase. Maximal phosphorylation activity was found at pH 7 (125.4 ± 6.7 U/mg) whereas lower activities were observed at pH 6 (81.3 ± 6.7 U/mg), pH 8 (116.8 ± 6 U/mg) and pH 9 (108.5 ± 3.3 U/mg)

*E. histolytica* fructokinase was produced at an estimated 30 % of the soluble protein in *E. coli* (Fig. 1), and about 1 μg of recombinant protein per microliter of eluate could be purified by nickel chelate affinity chromatography under native conditions. For measurements of fructokinase activity, the ADP generated was measured by coupled pyruvate kinase and lactate dehydrogenase reactions, and the decrease of NADH was examined spectrophotometrically at 340 nm. The activity of the fructokinase was MgCl_2_ dependent, with an optimum concentration of 10 mM (used in all experiments); no activity was observed with the addition of CaCl_2_. A slightly diminished fructokinase activity of 83 % was detected with 10 mM MnCl_2_ instead of MgCl_2._ The activity was rising up to a substrate concentration of 1 mM (108.5 U/mg; Fig. 3a, Table 1) and dropping to about half of the maximum at 5 mM substrate concentration. Calculated *V*_max_ of *E. histolytica* fructokinase at 37 °C was 131.3 ± 8.1 U/mg protein, and *K*_m_ for fructose was 0.156 ± 0.032 mM (Fig. 3b). Maximal phosphorylation activity was found at pH 7 (125.4 ± 6.7 U/mg), used in all assays. Lower activity was observed at pH 6 (81.3 ± 6.7 U/mg), pH 8 (116.8 ± 6 U/mg) and pH 9 (108.5 ± 3.3 U/mg) (Fig. 3c).

**Table 1:** Activity of the recombinant *E. histolytica* fructokinase using different substrates at various concentrations

	Activity (μmoles/min/mg)	Turnover (molecules/s)
Fructose (mM)		
5	50.5	27.5
1	108.5	59.1
0.5	106.5	58.0
0.1	53.1	29.0
0.05	27.1	14.8
0.01	5.3	2.9
0.005	0.0	0.0
Mannose (mM)		
5	12.5	6.8
1	2.5	1.4
0.5	0.0	0.0
Galactose (mM)		
5	0.0	0.0
Glucose (mM)		
5	0.0	0.0

Next, the activity of the recombinant enzyme with glucose, mannose, and galactose was tested. Limited activity with mannose but no activity with glucose or galactose was observed (Table 1). Moreover, the temperature optimum of *E. histolytica* fructokinase was at 37 °C (data not shown).

Potentially, the fructokinase reaction can generate fructose 1-phosphate or fructose 6-phosphate. To test for the activity generating fructose 6-phosphate, a coupled assay including glucose-6-phosphate isomerase and glucose-6-phosphate dehydrogenase was performed and the formation of NADPH during the latter reaction was measured by spectrophotometry. The calculated activity at 1 mM fructose concentration was 26.3 ± 1.1 U/mg protein with a turnover number of 14.3 ± 0.6 molecules per second, demonstrating the 6-phosphate forming activity of *E. histolytica* fructokinase.

### Fructokinase activity is elevated in lysates from fructose-adapted *E. histolytica* trophozoites

Fructokinase activity was examined in *E. histolytica* lysates via measurements of NADPH formation. In lysates of amoebae adapted to fructose, the measured fructokinase activity was 3-fold higher than in control amoebae. The calculated activity in fructose-adapted trophozoites was 12.3 ± 1.5 U/mg protein; the turnover number was 6.7 ± 0.8 molecules per second. Control amoebae grown in glucose showed a fructokinase activity of 3.9 ± 1.8 U/mg and a turnover number of 2.1 ± 0.5. So, the level of enzyme activity had risen more than the mRNA level, indicating additional post-transcriptional regulation.

### *E. histolytica* fructokinase localizes to the cytoplasm of trophozoites

**Fig. 4:**
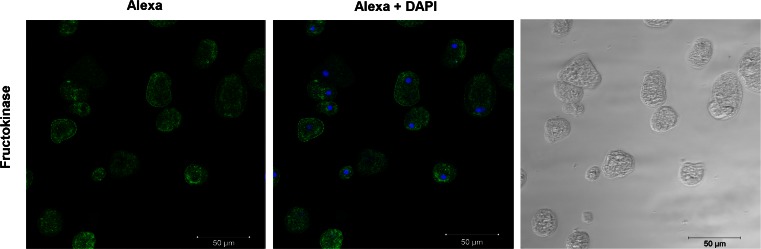
Localization of *E. histolytica* fructokinase. Paraformaldehyde-fixed trophozoites (*right panel*) were probed with a mouse serum against recombinant *E. histolytica*, and bound antibodies were visualized with Alexa Fluor 488 anti-mouse IgG (*left panel*). Nuclei were stained with DAPI (*middle panel*). No staining was seen when the trophozoites were stained with pre-immune serum (data not shown)

Confocal immunofluorescence microscopy was used to test the cellular localization of the fructokinase. A mouse serum was raised against the recombinant enzyme, and antibody binding was visualized with secondary anti-IgG antibodies labeled with Alexa 488 fluorescent dye. For staining of the nucleus, DAPI was used. Cytoplasmic localization was observed in amoebae stained with the fluorescent dye (Fig. 4), controls showed no staining (data not shown).

## Discussion

Only limited information exists about the response of *E. histolytica* to a lack of nutrients or changes in nutrient supply. Short-term (12 h) glucose starvation of trophozoites increased target cell lysis; moreover, the virulence of the trophozoites in a hamster liver abscess model was augmented. The lysine-rich protein KRiP1 was found to play an important role in this augmentation of virulence (Tovy et al. 2011). Deprivation of cysteine, a normal medium component, led to drastic changes in the metabolism of trophozoites (Husain et al. 2010). So far, it is not known, however, if this also increases their virulence.

Active glycolysis in *E. histolytica* was associated with uptake of oxygen. This respiration was maximally stimulated by glucose (100 %), much less by galactose (68 %), and even less by fructose (30 %) (Weinbach and Diamond 1974). Nevertheless, in this study, we showed that the trophozoites could be switched from glucose to fructose medium without any problems, and we studied fructokinase as the tool to metabolize fructose.

In the annotated *E. histolytica* genome database, there is a single fructokinase gene. The intronless 885 bp gene codes for a protein of 294 residues with a predicted molecular mass of 32.8 kDa and an isoelectric point of 5.87. Due to its association with bacterial sequences in the phylogenetic tree, the *E. histolytica* fructokinase gene was among the 96 candidates for lateral gene transfer (Loftus et al. 2005) and remained one up to this date with the availability of many more genomes (Grant and Katz 2014). All *Entamoeba* spp. sequenced to this date possess homologs of the fructokinase gene, so the event of lateral gene transfer may have been an early one. Interestingly, the single *E. histolytica* galactokinase gene is also most similar to bacterial galactokinase genes.

The upstream and downstream flanking regions of the *E. histolytica* fructokinase gene were retrieved from the AmoebaDB Database (http://amoebadb.org/amoeba/) and were found to be extremely A/T-rich (83–84 %) and short, only 77 bp to the neighboring upstream gene transcribed from the same strand, and only 64 bp to the downstream gene transcribed from the opposite strand. Whereas, expectedly, no putative signal peptide sequence, transmembrane domain, or glycosylation site were found in the deduced protein sequence; the neural network-based program NetPhos 2.0 (Blom et al. 1999) predicted the amino acid residues Ser128, Ser134, Ser146, Ser169, Ser177, Ser260, Ser273, Thr69, Tyr44, and Tyr220 as possible phosphorylation sites (score >0.8, cutoff >0.5). A high number of predicted phosphorylation sites is quite usual for *E. histolytica* proteins which corresponds to the large number of predicted protein kinases in this organism (Loftus et al. 2005).

In other protist parasites such as the trypanosomatids, *Plasmodium* spp., *Giardia intestinalis*, and *Trichomonas vaginalis*, no fructokinase gene was annotated or purified and characterized biochemically. Of course, this does not exclude such an activity by other sugar kinases. Two older studies on *Leishmania* spp. (Kreutzer and Christensen 1980) and on *Trypanosoma* spp. (Kreutzer and Sousa 1981) report the detection of a fructokinase activity by isoenzyme electrophoresis. Mertens and Müller (1990) described a fructokinase in *Tritrichomonas foetus*, as well as a separate glucokinase. The *T. foetus* fructokinase was dependent on ATP, with *K*_m_ values of 0.2 mM for fructose and 0.081 mM for ATP.

The *E. histolytica* fructokinase belongs to the large ribokinase family which consists of carbohydrate kinases of various specificities including fructokinases, phosphofructokinases, ribokinases, glucokinases, ketohexokinases such as ketodeoxygluconate kinase, and adenosine kinases (Bork et al. 1993). Several of these (Fuse et al. 2013; Caescu et al. 2004; But et al. 2012; Fennington and Hughes 1996; Perez-Cenci and Salerno 2014; Qu et al. 2004) are listed in Table 2, together with the *E. histolytica* fructokinase and few more bacterial fructokinases of the ROC type (Titgemeyer et al. 1994; Nocek et al. 2011; Thompson et al. 1991; Sato et al. 1993; Scopes et al. 1985). A sequence comparison of the mentioned ribokinase-like fructokinases is shown in Fig. S1. On one hand, similarities are obvious, especially in some fully conserved regions; on the other hand, significant divergence exists. As an example, the *E. histolytica* and *Prevotella intermedia* (Fuse et al. 2013) sequences are 46.8 % identical on the amino acid level.

**Table 2:** Comparison of bacterial fructokinases to the *E. histolytica* enzyme

Organisms	Kinase family	*V*_max_ (U/mg)	*K*_m_ (mM)	Substrate specifity	Cations	pH optimum	Temperature optimum (°C)	Reference
*Entamoeba histolytica*	Ribokinase	131	0.156	Fructose, mannose	Mg^2+^ > Mn^2+^	7–8	37	This work
*Bifidobacterium longum*	Ribokinase	0.84	0.739	Fructose	?	6	50	Caescu et al. 2004
*Methylomicrobium alcaliphilum*	Ribokinase	141	0.26	Fructose	Mn^2+^	9	60	But et al. 2012
*Prevotella intermedia*	Ribokinase	?	?	Fructose	Mg^2+^	?	?	Fuse et al. 2013
*Rhizobium leguminosarum*	Ribokinase	31	0.31	Fructose	Co^2+^ > Mg^2+^ > Cd^2+^ > Mn^2+^ > Ca^2+^	8	25	Fennington and Hughes 1996
*Synechococcus* sp.	Ribokinase	?	?	Fructose, maltose (trace)	Mg^2+^	7.5	30	Perez-Cenci and Salerno 2014
*Thermococcus litoralis*	Ribokinase	730	2.3	Fructose	Mg^2+^ > Mn^2+^ > Co^2+^	7.5–8	80	Qu et al. 2004
*Bacillus subtilis*	ROK	178	0.38	Fructose	?	8	25	Nocek et al. 2011
*Lactococcus lactis*	ROK	190–200	0.31	Fructose, mannose	Mg^2+^ > Co^2+^ > Fe^2+^ > Mn^2+^ > Ni^2+^ > Zn^2+^ > Cd^2+^	7–8	25–40	Thompson et al. 1991
*Streptococcus mutans*	ROK	20	0.77	Fructose, mannose	Mg^2+^	7.4	25	Sato et al. 1993
*Zymomonas mobilis*	ROK	350	0.7	Fructose, mannose (trace)	?	8	25	Scopes et al. 1985

Table 2 shows some more similarities between the related fructokinases from *E. histolytica* and various bacteria. For instance, the *E. histolytica* enzyme also displayed a limited activity with mannose besides the major fructokinase activity (Table 1). Mannose-phosporylating activity was also observed in the ROC-type fructokinases from *Lactococcus lactis* (Thompson et al. 1991) and *Streptococcus mutans* (Sato et al. 1993), and a trace activity was found in *Zymomonas mobilis* (Scopes et al. 1985). The maximum fructokinase activity of the *E. histolytica* enzyme was observed at 1 mM fructose and decreased significantly at 5 mM substrate concentration (Fig. 3a). Such substrate inhibition was also observed in the fructokinase from potatoes (Renz and Stitt 1993). *E. histolytica* fructokinases and all the bacterial fructokinases mentioned in Table 2 required bivalent ions for their activity, preferentially Mg^2+^ with the exception of *Methylmicrobium alcaliphilum* fructokinase which required Mn^2+^ for its activity (But et al. 2012). In total, the *V*_max_ of *E. histolytica* was about average compared to the bacterial enzymes, but the *K*_m_ was lowest, allowing activity at lower fructose concentrations. Moreover, most species showed similar pH optima as found for *E. histolytica*; only the *M. alcaliphilum* enzyme (But et al. 2012) had a basic pH optimum. The temperature optimum of the *E. histolytica* fructokinase corresponded to the temperature of the human host; the enzyme with the highest activity from *Thermococcus litoralis* (Qu et al. 2004) had the highest optimum temperature of 80 °C.

Recombinant *E. histolytica* fructokinase produced fructose 6-phosphate. On one hand, this is a direct intermediate of classical glycolysis and can be used for the generation of energy. On the other hand, we noted that fructose 6-phosphate can be converted in one step to glucosamine 6-phosphate by a glucosamine–fructose-6-phosphate aminotransferase (candidate XP_650078) followed by acetylation by a glucosamine-6-phosphate *N*-acetyltransferase (candidates XP_655194, XP_649522, and XP_648703). The resulting *N*-acetylglucosamine 6-phosphate can serve as a direct precursor of chitin in the *E. histolytica* cyst wall.

The mRNA encoding this enzyme was significantly upregulated in amoebae adapted to fructose, as well as in trophozoites cultured with fructose for 2 and 4 h, respectively (Fig. 2). However, the highest fructokinase mRNA upregulation, which was observed after 4 h, was only as high as about 2-fold compared to amoebae grown in normal medium. Thus, the parasite responded to chemical stress only with rather moderate changes of mRNA expression. The fructokinase activity measured in extracts from fructose-adapted *E. histolytica* was about 3-fold higher than in control extracts, pointing to some post-transcriptional regulation. Interestingly, the effect of fructose on the fructokinase expression in *E. histolytica* was comparable to what was found in bacteria before. So, in *Zymomonas mobilis*, the mRNA level increase was 3-fold whereas the fructokinase activity increased about 2-fold when the bacteria were grown on fructose instead of glucose (Zembrzuski et al. 1992).

This work represents the first biochemical study on the *E. histolytica* fructokinase. The enzyme allows the trophozoites to grow on fructose which may be more abundant in the human colon than glucose. In vitro, *E. histolytica* adapted to fructose media without any problems and modestly upregulated fructokinase expression on the levels of mRNA and enzyme activity. So, taken together, the *E. histolytica* fructokinase is a new example for an important metabolic activity, for which the gene was most likely acquired by lateral gene transfer.

## Electronic supplementary material

Below is the link to the electronic supplementary material.

ESM 1 (DOC 31 kb)
