# Supplementary material for: Molecular and biochemical characterization of Entamoeba histolytica fructokinase
Source: Parasitol Res. 2015 Feb 21;114(5):1939–47. doi: 10.1007/s00436-015-4383-5 (PMC4412284; doi:10.1007/s00436-015-4383-5)
Supplement: Supplementary file 1 — (DOC 31 kb) [file 436_2015_4383_MOESM1_ESM.doc]

10 20 30 40 50 60 70 80

Enthis ----MNHKKIKVAGIGEVVWDCFGDV-KKQGGAPCNFAMHMAQFGFESYAFIAVGNDELGKRSLEIIHSFGVQTIDPVVD--YETSTVII

Biflon ---MPNTTKPSVVAVGELLWDMLPDG-RKPGGAPANFIYHVAQNGVQGTAVTAVGNDDLGRDLVSILADNGVNVAAQVND--YPTGVTAV

Rhileg --MILCCGEALIDMLPRDTTLGEKGFAPYAGGAIFNTAIALGRLGIPTAFFTGIADDMMGEILLETLKASNVDYSPCAITP-RPSTIAFV

Synsp -------MAKNIVIFGEVLWDIFPDNDRKLGGAPFNVAWHLKAFGANPLLISRIGTDDLGATIQTKMQAWQMTLAGLQIDKIHPTGTVKV

Thelit MIYAIGEILIDFIAKEEGKLKDVREFEKHPGGAPANVVVGLRRLGAKSALISKVGDDPFGEFLIEELKKERVETKYIIKDTNKHTGIVFV

Preint -------MKQYIVGLGEALWDCLPEG-KKIGGAPANFAFHAGQFGHDSIAISAIGNDKLGEETLAEFDKKHVNYLMPVVD--YPTGTVQV

Metalc ------MNKNPITIFGEVLFDHFPDGSRVLGGAPFNVAWHLQAFRQEPHFISRVGQDATGDSVIAAMHKWGMDMSGLQRDSRHPTGVVDI

. . *** * . :. * * : : : . :

90 100 110 120 130 140 150 160

Enthis TLHNG-IPSYNVKLNVAWDHLKLTDSIIEKAKELDAVCFGTIAQRSEE-TRKSIIQFLKLMKPNSFKVFDVNLRQHFYN------DDIIQ

Biflon RLDDNGIPEYDVVKGVAWDHIEFTDNVRALVRAADAICYGTIAAREAWGTRDTIYQMIDAARPDAIRLFDINIRSGFVN------KEVIT

Rhileg KLVN-GQATYAFYDEGTAGRMITTADLPDLGDDCEALHFGAISLIPSPCG-ETYEALLDREAASRVISLDPNIRPGFIKDKP-SHMARIK

Synsp TLEK-GQPQYEITADCAYDFINSQQFPILEDKFWLYHGSLALRNAVSQASFRALQQRAD-Q-----IFFDVNLRQPWWT------LETIA

Thelit QLIG-AKPEFILYDGVAYFNLRKEEIQWDFMRDAELLHFGSVLFARE-PSRSTVFEVLRAVKGKVPISYDVNIRLDLWRGREKEMLKDIE

Preint TLDEAGIPTYEIKEGVAWDNIPFTSEIEEVAKNCRAVCFGSLAQRSSV-SRNTIQQFLDATPDDCLKIFDINLRQNFYN------ENIIR

Metalc VIEQ-GEPAYTIVPEQAYDYIDEDELQDTDRPGLLYHGTLSLRQPVSRAALDVLKDAHKGR-----IFMDVNLREPWWQ------KDQVL

: . : . : : :: * *:* :

170 180 190 200 210 220 230 240

Enthis ESLSLSNIVKMSDEEIQEVGKACGFQGNDLEILKQIHH------QYHLKYSLLTLG--EKGSYVYDGTNEIFCEPTKVNVVNTVGAGDSF

Biflon RFLEGATVLKINDEELPIVANLFGLDSPGKDIRSQQRAMRTLCEMFNLDVAILTAG--DAYSIVMGHDEISILPTPQVEVADTVGAGDSF

Rhileg RMAAKSDIVKFSDEDLDWFGLQGDHDALAAHWLNHG-----------AKLVVITKG--AEGASGYTKDRKVTVPSERVTVVDTVGAGDTF

Synsp SALAASQYVKLNTEELRLLTPEFSSTNLAIDHLLNQ---------NSLRHIILTAG-EAGASLYTQGDRQQISPLQNTTVVDTVGAGDAF

Thelit EALKLADIVKIGDGELEYLNKNGIALED-----------------FNFALVAITRG--AEGSTIIHKDIRVDVPSFKVEPVDTTGAGDAF

Preint NSLKQCNILKINDEELVAIGRIFGYPGLDMENKCWLLIG-----KYNLDMLVLTCG--VNGSYVFAPNLVSFQETPKVDVADTVGAGDSF

Metalc EWLGQADWVKLNHHELAALYPVSGDLKADMRRFVEL---------YRLQGLIVTSGKQGAFATDHQGVSCRVTPGEIAQVIDTVGAGDAF

. :*:. :: . :* * : . :*.****:*

250 260 270 280 290

Enthis TAIFVGSILKG----------KSIEQAQKLASKVASYVCTQDSAMPKLTQELLSELK-----------------------

Biflon SGAFLAYLLRG----------FAIPAAHRRAVDVSAFVCSQSGAWPRYPQELREQQPHGLSSIGSGNPLQ----------

Rhileg DAGILASLKMDN-----LLTKRQVASLDEQALRNGPDPRRQSRRRHRLPRRRQSTLGARDWSLRLEQDSDPHPPDDTFSP

Synsp CSICLLGLMND----------WPSVLTLERAQAFASAIVGIRGAVSEDPRFYQPFIQAWRL-------------------

Thelit MAALLASLFYMGKLDILEFSKEELKELGSFANLVAALSTTKRGAWSVPSLEEVLKHRKFSFLP-----------------

Preint TGTFAAAILAG----------KCIPEAHKLAVEASAYVCTQNGAMPILPKELIEKAK-----------------------

Metalc ASVMLLGLNLD----------WPLQITMERAQAFASAMVGQRGATVRDPVFYEPFIAAWDLD------------------

. : * .. .

**Fig. S1** Comparison of fructokinases belonging to the ribokinase group from various species. The residues were numbered according to the *E. histolytica* sequence. Enthis (XP_652087), *E. histolytica*; Biflon (WP_032746522), *Bifidobacterium longum* (Caescu et al. 2004); Rhileg (P42720), *Rhizobium leguminosarum* (Fennington and Hughes 1996); Synsp (CDL67996), *Synechococcus* sp. (Perez-Cenci and Salerno 2014); Thelit (AF307053), *Thermococcus litoralis* (Qu et al. 2004); Preint (WP_028906351), *Prevotella intermedia* (Fuse et al. 2013); Metalc (YP_004915908), *Methylomicrobium alcaliphilum* (But et al. 2012).
